# C-Reactive Protein and White Blood Cell Count as Triage Test Between Urgent and Nonurgent Conditions in 2961 Patients With Acute Abdominal Pain

**DOI:** 10.1097/MD.0000000000000569

**Published:** 2015-03-06

**Authors:** Sarah L. Gans, Jasper J. Atema, Jaap Stoker, Boudewijn R. Toorenvliet, Helena Laurell, Marja A. Boermeester

**Affiliations:** From the Department of Surgery (SLG, JJA, MAB); Department of Radiology(JS), Academic Medical Centre, Amsterdam; Department of Surgery (BRT), Ikazia Hospital, Rotterdam, the Netherlands; and Department of Surgery (HL), Mora Hospital, Mora, Sweden.

## Abstract

The purpose of this article is to assess the diagnostic accuracy of C-reactive protein (CRP) and white blood cell (WBC) count to discriminate between urgent and nonurgent conditions in patients with acute abdominal pain at the emergency department, thereby guiding the selection of patients for immediate diagnostic imaging.

Data from 3 large published prospective cohort studies of patients with acute abdominal pain were combined in an individual patient data meta-analysis. CRP levels and WBC counts were compared between patients with urgent and nonurgent final diagnoses. Parameters of diagnostic accuracy were calculated for clinically applicable cutoff values of CRP levels and WBC count, and for combinations.

A total of 2961 patients were included of which 1352 patients (45.6%) had an urgent final diagnosis. The median WBC count and CRP levels were significantly higher in the urgent group than in the nonurgent group (12.8 ×10^9^/L; interquartile range [IQR] 9.9–16) versus (9.3 ×10^9^/L; IQR 7.2–12.1) and (46 mg/L; IQR 12–100 versus 10 mg/L; IQR 7–26) (*P* < 0.001).

The highest positive predictive value (PPV) (85.5%) and lowest false positives (14.5%) were reached when cutoff values of CRP level >50 mg/L and WBC count >15 ×10^9^/L were combined; however, 85.3% of urgent cases was missed.

A high CRP level (>50 mg/L) combined with a high WBC count (>15 ×10^9^/L) leads to the highest PPV. However, this applies only to a small subgroup of patients (8.7%). Overall, CRP levels and WBC count are insufficient markers to be used as a triage test in the selection for diagnostic imaging, even with a longer duration of complaints (>48 hours).

## INTRODUCTION

The acute abdomen represents a major diagnostic challenge at the emergency department (ED). Up to 10% of all patients at the ED present with complaints of acute abdominal pain.^1,2^ Underlying causes vary between mild and self-limiting conditions to conditions requiring urgent treatment.^3–5^

Clinical evaluation is often insufficient to correctly diagnose the underlying cause. The accuracy of clinical assessment (history and physical examination and laboratory evaluation) has been reported between 47% and 76%.^3,6–8^ Management based on clinical assessment alone can result in overtreatment or cause delay of vital treatment. Imaging modalities such as ultrasound and computed tomography (CT) have been increasingly used to enhance diagnostic accuracy.^1,3,9^

Studies have demonstrated that the use of imaging leads to a decrease in missed urgent conditions and false-positive diagnoses. Imaging also increases diagnostic certainty and changes management decisions.^3^ However, the increased use of imaging also has downsides. The hospital costs rise exponentially, patient throughput at the ED is protracted, and, in case of CT, patients are exposed to ionizing radiation and contrast agents.^1,10^

A timely and accurate diagnosis leads to improved outcomes in case of urgent conditions.^3^ It is therefore essential to rapidly distinguish between patients with an urgent condition and those with a nonurgent condition. Ideally, clinical evaluation would lead to an accurate selection of patients with an urgent condition, in whom immediate imaging is required without exposing patients with a nonurgent condition to unnecessary imaging.

The inflammatory markers, C-reactive protein (CRP) and white blood cell (WBC) count, are routinely determined as part of the workup of patients with an acute abdomen. These markers rise rapidly in response to various infectious and inflammatory conditions.^11–13^ However, elevated levels are nonspecific and their diagnostic accuracy for a specific diagnosis is low. CRP and WBC count could be helpful in discrimination between urgent and nonurgent conditions, and to function as a triage test in the selection of patients for immediate additional imaging and the identification of patients with nonurgent conditions in whom no immediate imaging is required. With a longer duration of complaints, the discriminative power of CRP levels and WBC count may increase.

The aim of this study is to assess the value of CRP levels and WBC count in differentiating suspected urgent conditions—requiring immediate imaging workup and further treatment—from suspected nonurgent conditions—not requiring immediate workup—in patients with acute abdominal pain at the ED.

## MATERIAL AND METHODS

### Study Selection and Patients

Three large prospective cohort studies of patients with acute abdominal pain at the ED were identified by a literature search.^3,5,14^ Principal investigators of eligible studies were invited to participate by e-mail. The investigators were asked to share their complete dataset in original format with complete, anonymous data. All received data were carefully examined for inconsistencies between the data and their original studies. Received data were converted and recoded into a uniform format. A separate data dictionary of each study was requested to prevent errors in conversion of the individual studies to one uniform format. Issues or inconsistencies were checked with the principal investigators. Full study design of the included studies is described in the original publications.^3,5,14^ All studies were approved by the institutional review board of the initiating center.

In each study, a final diagnosis had been assigned to patients by an expert panel. The final diagnosis was based on all available data, including at least 3 months of follow-up and, if available, histopathology, imaging, or surgery reports.

After harmonization of the databases, only the adult patients (>18 years) of each study were selected for inclusion. A new variable was created in order to classify the final diagnosis into urgent and nonurgent conditions, based upon the classification proposed by Lameris et al.^3^ Urgent conditions were defined as conditions requiring treatment within 24 hours. Duration of symptoms was categorized into 3 categories: <24, 24–48, and >48 hours. Patient data were only included if CRP levels or WBC counts were available.

### Study Quality Assessment

The quality of the included studies was assessed from their original publication using the QUADAS-2 checklist.^15^ Completeness of datasets was assessed and described based on availability of data on CRP levels and WBC counts, final diagnosis, and duration of complaints. Review manager was used to summarize the results of the QUADAS-2 assessment.

### Statistical Analysis

Data analysis was performed according to the Preferred Reporting Items for Systematic Reviews and Meta-Analyses statement.^16^ The baseline characteristics were analyzed using descriptive statistics. Continuous variables were tested for normality using the Shapiro–Wilk test. Group differences between urgent and nonurgent groups were tested using the Mann–Whitney U test. Nonnormally distributed continuous data were expressed as median and interquartile range (IQR). Probability (*P*) values were considered significant at a cutoff point of 0.05. A CRP level of >10 mg/L and a WBC count of >10 ×10^9^/L were considered elevated above the reference standard. CRP levels and WBC count were plotted for urgent and nonurgent groups in box plots to demonstrate their distribution.

The values of CRP levels and WBC were categorized into several clinically relevant and applicable cutoff values. We constructed 2 × 2 contingency tables for each of the cutoff values of CRP and WBC in the database. The sensitivity and specificity of CRP and WBC for detecting urgent conditions were calculated by comparing the results of the cutoff scenarios with the final diagnoses. The percentage of missed urgent cases (1-sensitivity), the percentage of false positives (false positives/all positives), the positive predictive values (PPVs) (true positives/all positives), and negative predictive values (NPVs) (true negatives/all negatives) were calculated using the contingency tables. The false positives are patients with a final nonurgent diagnosis and elevated CRP level or WBC count above the cutoff. The missed urgent cases are the patients with a final urgent diagnosis and normal CRP levels or WBC count. The discriminatory value of CRP and WBC was analyzed by calculating the area under the receiver-operating curve (AUC). An AUC of >0.80 was considered to indicate good discrimination. These analyses were repeated for each of the separate time categories (duration of complaints). All data were analyzed using SPSS 20.0 (SPSS Inc., Chicago, IL) and MedCalc for Windows 12.5 (MedCalc Software, Ostend, Belgium).

## RESULTS

### Study Characteristics

**FIGURE 1:**
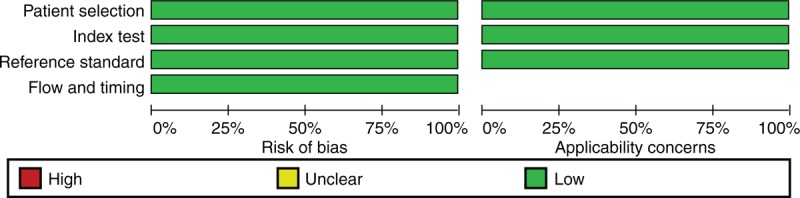
Overview of methodological quality of reporting of included studies according to the QUADAS-2 checklist.

Three large prospective cohort studies were included, comprising a total of 2961 adult patients presenting at the ED with acute abdominal pain. Two studies were performed in the Netherlands^3,14^ and 1 in Sweden.^5^ The study designs and baseline characteristics of the 3 cohorts were comparable (Table 1). The inclusion criteria differed between the studies. In 1 study,^3^ only patients were included when imaging was deemed necessary by the treating physician whereas the other 2 studies^5,14^ included all consecutive patients with acute abdominal pain. An overview of the quality of the included studies according to the criteria of the QUADAS-2 checklist is shown in Figure 1.

**TABLE 1:**
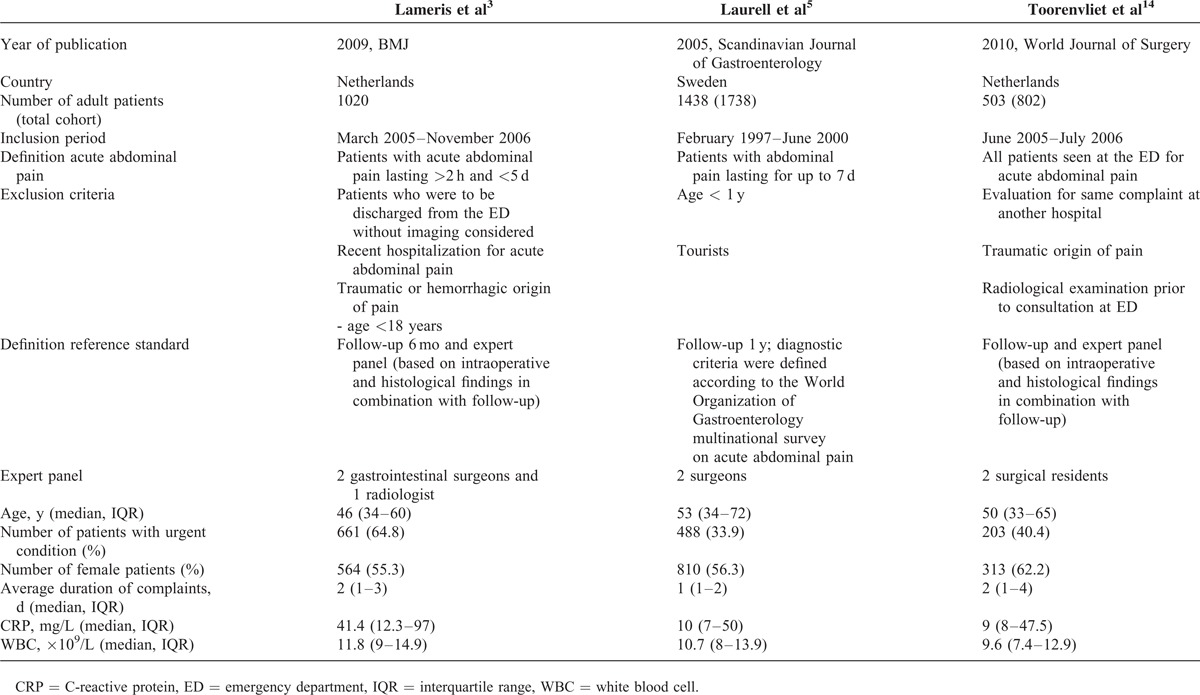
Characteristics of the Included Studies

### Baseline Characteristics

In 1352 patients (45.6%), the final diagnosis was classified as urgent and in 1609 patients (54.3%), it was classified as nonurgent (Table 2). The percentage of males was significantly higher in the urgent group (48.7%) compared with the nonurgent group (38.3%) (*P* < 0.001). The median age was 45.4 years (IQR 31.2–64.2) in the nonurgent group and 53.7 years (IQR 38.4–68.7) in the urgent group (*P* < 0.001). The median duration of pain was 1 day in both the groups (*P* = 0.469).

**TABLE 2:**
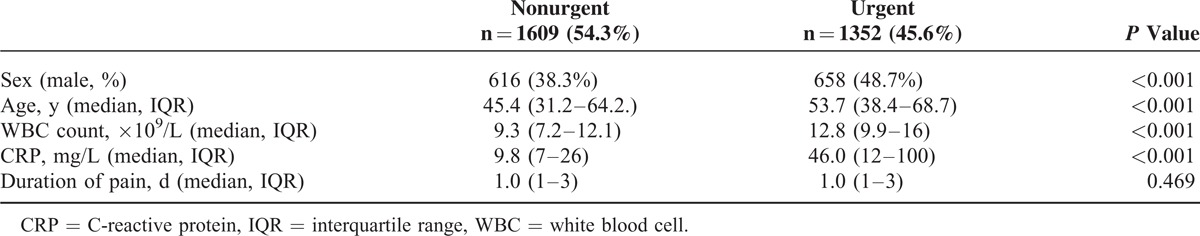
Characteristics of Patients Classified by Urgency

The most common urgent conditions were acute appendicitis (15.0%) and acute diverticulitis (8.4%) (Table 3). The most common nonurgent condition was nonspecific abdominal pain (24.6%) followed by gastrointestinal diseases (8.3%). Nonabdominal causes accounted for 1.2% of urgent causes and 3.1% of nonurgent causes. Malignancies were found in 1.7% of all patients. A gynecological cause (both urgent and nonurgent) was found in 3.6% of all patients and an urological origin in 7.1% of patients.

**TABLE 3:**
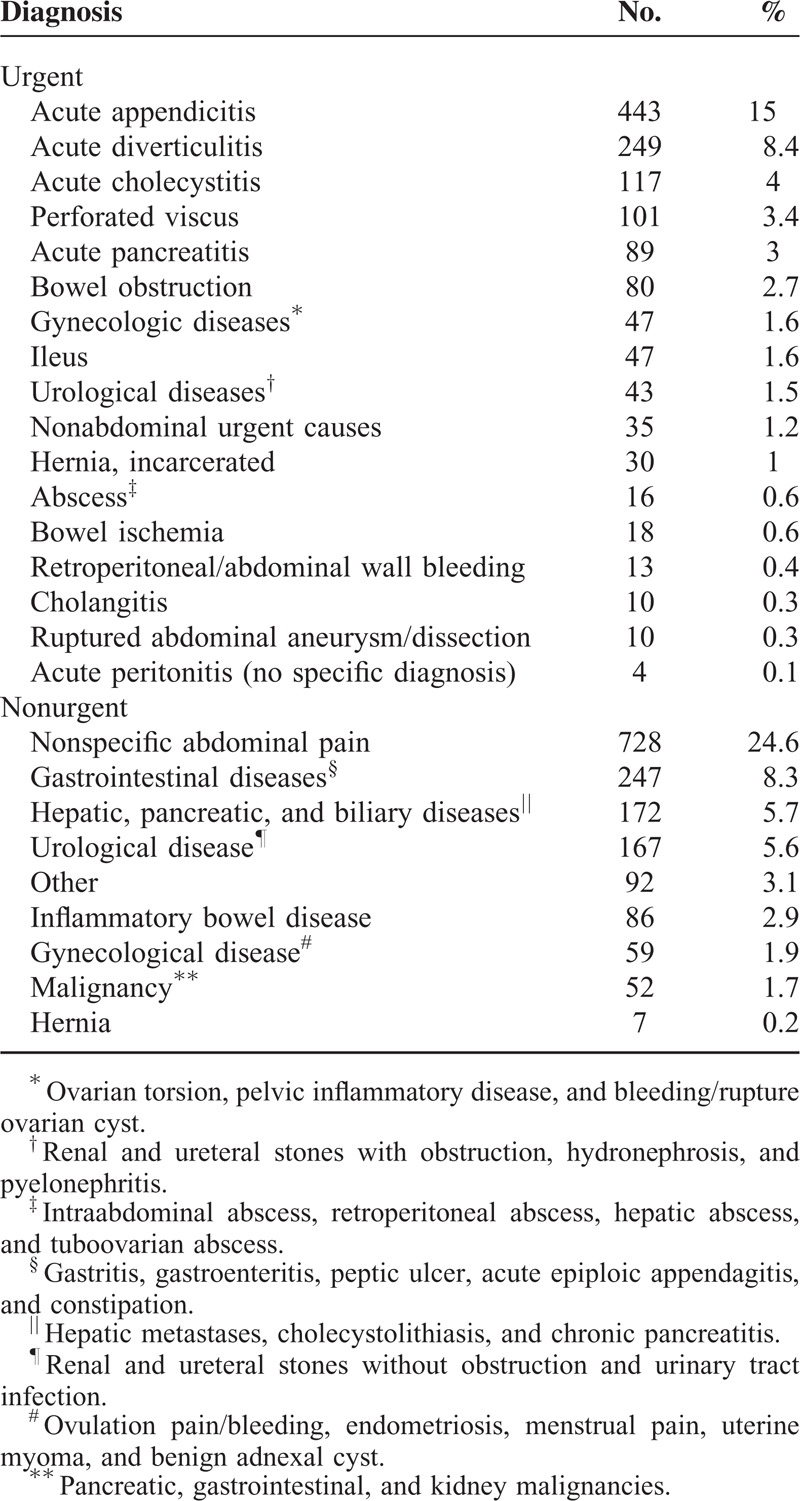
Final Diagnoses in 2961 Patients Classified by Urgency

### Distribution of CRP Levels and WBC Count in Urgent and NonUrgent Causes

**FIGURE 2:**
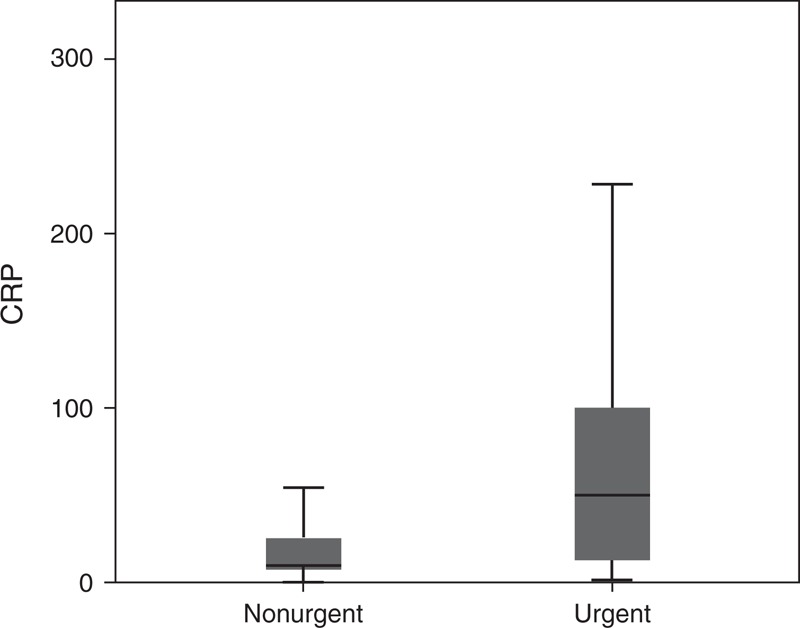
Box plot of the distribution of values of C-reactive protein in patients with urgent versus nonurgent diagnoses (*P* < 0.001).

**FIGURE 3:**
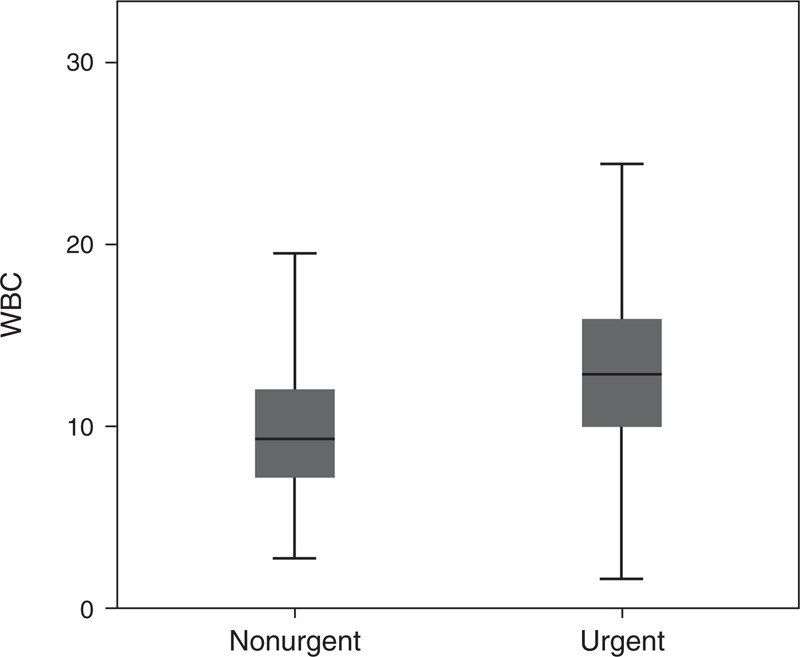
Box plot of the distribution of values of white blood cell count in patients with urgent versus nonurgent diagnoses (*P* < 0.001).

In 2783 of the 2961 patients (93.9%), CRP levels had been determined during ED evaluation, and WBC count in 2636 patients (89.0%). For 2458 of 2962 patients (82.9%), both CRP levels and WBC count were available. The distribution of CRP levels and WBC count is depicted in Figures 2 and 3. The median CRP and WBC values were raised above the reference value in patients in the urgent group, whereas in the nonurgent group, the median CRP and WBC values were within the normal range (Table 2). The median CRP level was significantly higher in the urgent group, 46.0 mg/L (IQR 12–100) compared with 9.8 mg/L (IQR 7–26) in the nonurgent group (*P* < 0.001). The median WBC count was also significantly higher in the urgent group (12.8 ×10^9^/L; IQR 9.9–16) compared with the nonurgent group (9.3 ×10^9^/L; IQR 7.2–12.1; *P* < 0.001) (Table 2).

### Diagnostic Accuracy

Table 4 depicts the diagnostic accuracy of several cutoff values of CRP levels, WBC count, and their combinations. CRP had an area under the curve of 0.721 and WBC count of 0.712. CRP was elevated (CRP >10 mg/L) in 56.2% of the patients (1565/2783). Using an elevated CRP level as cutoff resulted in a sensitivity of 76.9% (95% confidence interval [CI] 74–79) and a specificity of 61.4% (95% CI–64). This cutoff value would lead to 36.9% false-positive diagnoses and 23.1% missed urgent diagnoses. Raising the cutoff value up to a CRP >150 mg/L increased the specificity up to 95.8% (95% CI 95–97), but also led to a decreased sensitivity of 15.7% (95% CI 14–18) and therefore 84.3% missed urgent diagnoses. It is of note that in only 9.4% of all 2783 patients, CRP values were elevated above 150 mg/L.

**TABLE 4:**
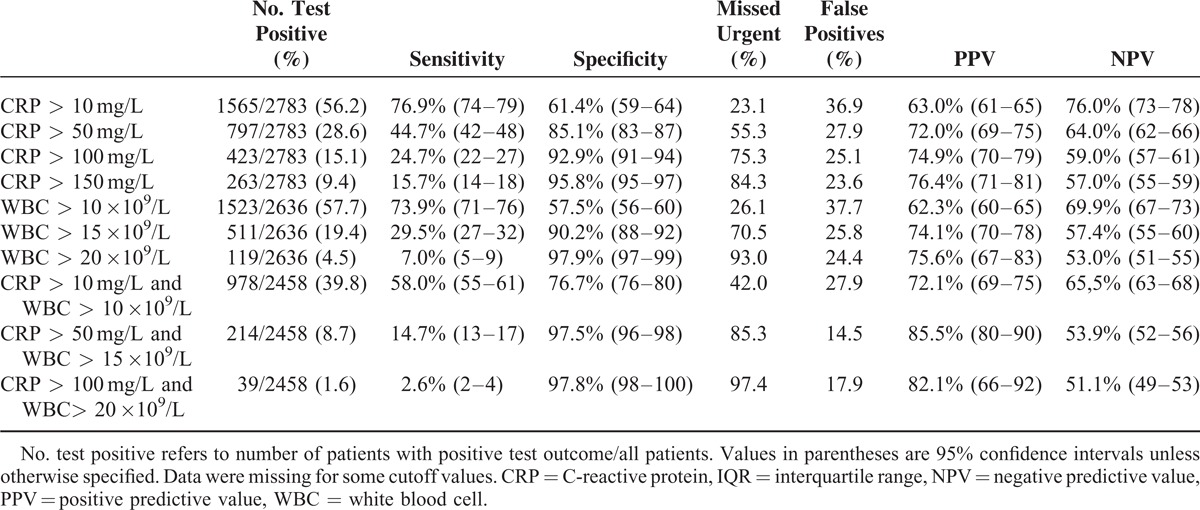
Discriminatory Accuracy of Different CRP and WBC Cutoff Values, and Combinations, for Urgent Versus Nonurgent Conditions

In 57.7% of patients, WBC count was elevated (1523/2636). An elevated WBC count (WBC >10 ×10^9^/L) resulted in a sensitivity of 73.9% (95% CI 71–76) and a specificity of 57.5% (95% CI 56–60). In 26.1%, an urgent diagnosis was missed and in 37.7%, the diagnosis was falsely positive. Raising the cutoff value up to a WBC count >20 ×10^9^/L resulted in a specificity of 97.9% (95% CI 96–98), but decreased sensitivity down to 7.0% (95% CI 5–9) leading to 93.0% missed urgent diagnoses and 24.4% false-positive diagnoses. In only 4.5% of all 2636 patients, the WBC count was raised >20 ×10^9^/L.

Combining cutoff values of CRP and WBC count increased both the PPV and the NPV. The combination of an elevated CRP level and WBC count (CRP >10 mg/L and a WBC count >10 ×10^9^/L) resulted in a sensitivity of 58.0% (95% CI 55–61) with a specificity of 76.7% (95% CI 76–80). This cutoff value led to 42.0% missed urgent diagnoses and 27.9% false-positive diagnoses. In 39.8% of patients, both the CRP level and the WBC count were elevated (978/2458). A combination of intermediate cutoff values (CRP >50 mg/L and WBC >15 ×10^9^/L) increased the specificity up to 97.5% (95% CI 96–98) and decreased sensitivity to 14.7% (95% CI 13–17). These values led to a PPV of 85.5% (95% CI 80–90) but with a high percentage of missed urgent cases (85.3%). In only 8.7% of patients, both the CRP level and the WBC count were higher than these cutoff levels.

Extreme values of CRP and WBC count (CRP >100 mg/L and WBC >20 ×10^9^/L) decreased the sensitivity even further down to 2.6% (95% CI 2–4) and increased the specificity up to 97.8% (95% CI 96–98). The percentage of missed urgent diagnoses remained unacceptably high (97.4%) with a PPV of 82.1% (95% CI 66–92). However, only in 1.6% of patients, both CRP and WBC count were severely elevated.

### Duration of Complaints

For each category of duration of complaints (<24, 24–48, and >48 hours), the median values of CRP and WBC count were significantly higher (*P* < 0.001) in patients with an urgent condition compared with patients with a nonurgent condition (Tables 5 and 6). The median levels of CRP increase in patients with a longer duration of complaints. The median levels of WBC remained the same, regardless of the duration of symptoms. The AUC for CRP was 0.695 for duration of complaints <24 hours, 0.698 for duration between 24 and 48 hours, and 0.756 for duration >48 hours. The AUC for CRP was significantly higher for duration >48 hours compared with a duration between 24 and 48 hours (*P* = 0.005. The AUC for WBC was 0.702 for duration of complaints for <24 hours, 0.716 for duration between 24 and 48 hours, and 0.725 for duration >48 hours. When comparing the AUCs between the categories of duration of complaints, there were no significant differences. The discriminatory value improved somewhat at a longer duration of complaints. CRP levels of >10 mg/L after >48 hours of complaints resulted in the highest sensitivity (91.0%). However, the associated specificity was only 47.0%, with a false-positive diagnosis of an urgent condition in 37.8% of cases. Tables 7 to 9 depict the discriminatory value of CRP levels and WBC count classified according to the duration of complaints.

**TABLE 5:**
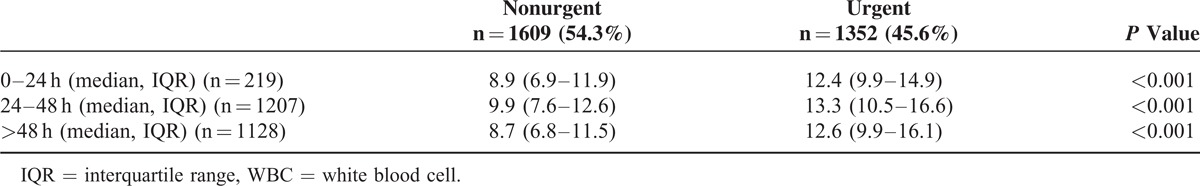
Distribution of WBC for Duration of Complaints in Urgent Versus Nonurgent Conditions

**TABLE 6:**
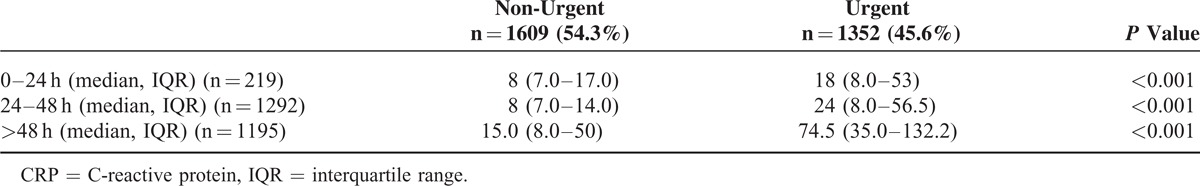
Distribution of CRP for Duration of Complaints in Urgent Versus Nonurgent Conditions

**TABLE 7:**
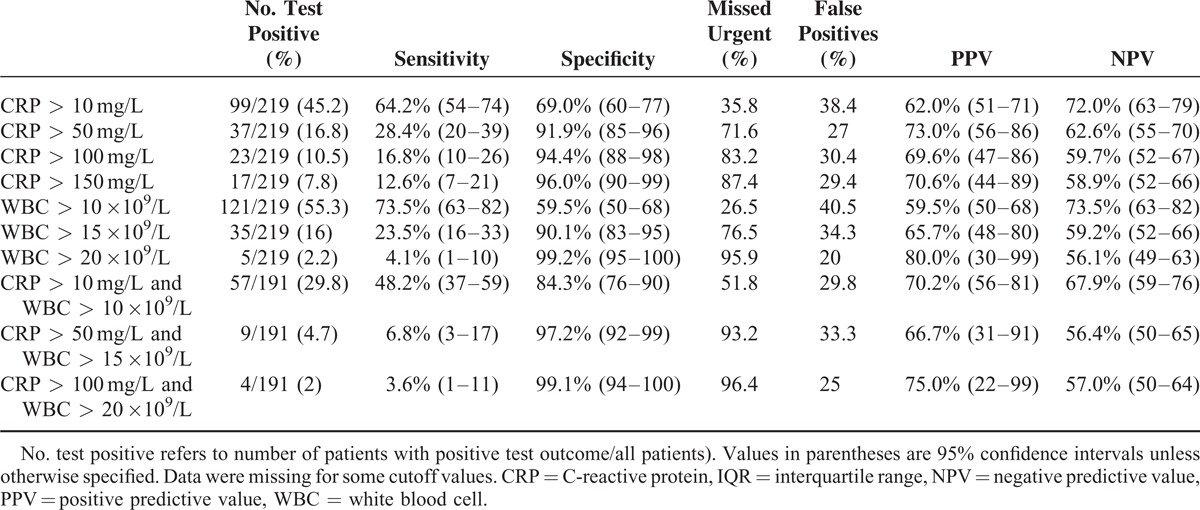
Discriminatory Accuracy of Different CRP and WBC Cutoff Values and Combinations With Duration of Complaints Between 0 and 24 h

**TABLE 8:**
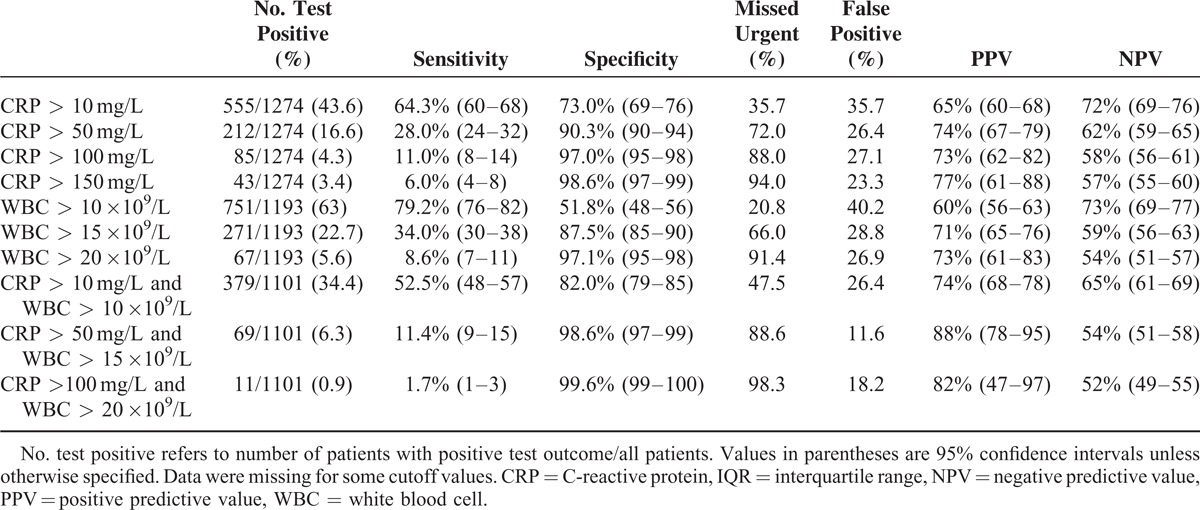
Discriminatory Accuracy of Different CRP and WBC Cutoff Values and Combinations With a Duration of Complaints Between 24 and 48 h

**TABLE 9:**
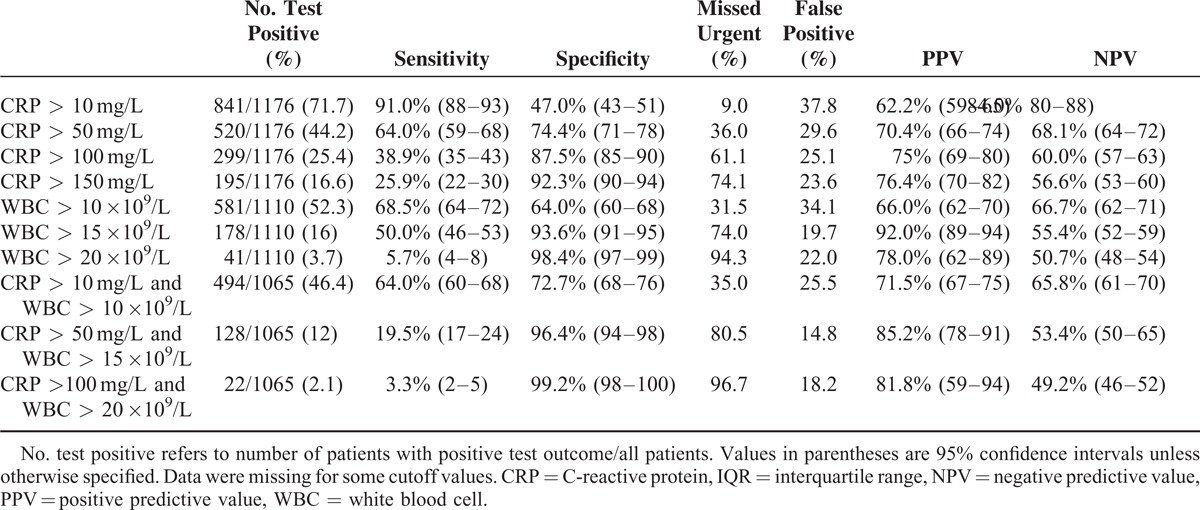
Discriminatory Accuracy of Different CRP and WBC Cutoff Values and Combinations With a Duration of Complaints of >48 h

## DISCUSSION

The discriminatory value of CRP levels and WBC count as single markers in differentiating urgent conditions from nonurgent conditions in patients with acute abdominal pain is low, even with an increased duration of symptoms (>48 hours). Overall, CRP levels and WBC count are insufficient markers to be used as a triage instrument in the selection for diagnostic imaging.

A CRP value or WBC count within the reference range does not rule out an urgent condition. Even in patients with an urgent final diagnosis, CRP and WBC count can be well within reference values, and vice versa even extreme values of CRP or WBC count do not guarantee the presence of an urgent condition. Although the median values of CRP and WBC count in patients with urgent conditions are significantly higher compared with values in patients with nonurgent conditions, there is no sufficient cutoff value that can adequately distinguish enough patients with an urgent condition. Higher cutoff values of CRP or WBC count lead to an unacceptably low sensitivity (high proportion of missed urgent cases) and high percentage of false-negative diagnoses. Intermediate cutoff values such as CRP >100 mg/L or WBC count >15 ×10^9^/L led to an unacceptably high percentage of false-positive rates ranging between 25.1% and 25.8%, respectively. An intermediate CRP level (>50 mg/L) combined with an intermediate WBC count (>15 ×10^9^/L) achieves the highest PPV, justifying diagnostic imaging in this subset of patients. This combination, however, misses the greatest proportion of urgent cases (85.3%) and only a small subset of patients (8.7%) meet both these cutoff levels. The value of CRP and WBC count as triage test in daily practice is limited.

Most studies analyzing the value of CRP levels and WBC count focus on a selection of patients such as patients with suspected acute appendicitis.^17,18^ These studies conclude that the laboratory values individually are weak discriminators but when combined with clinical parameters they achieve high discriminative powers. Studies analyzing the value of CRP levels and WBC count in patients with an acute abdomen report varying results. Some studies have reported that there is very little correlation between CRP values and the outcomes of the patient and that CRP alone is not useful in differentiating self-limiting conditions from causes that need surgery.^19,20^ Conversely, another study demonstrated that increasing levels of CRP predict positive findings on CT with increasing likelihood suggesting that inflammatory markers can be used in prioritizing patients for imaging.^10^

Given the disadvantages of imaging, a triage test discriminating between patients with an urgent condition, in whom additional imaging is justified, and patients without an urgent condition, in whom no emergency imaging is needed, would be extremely useful. An accurate triage test could prevent unnecessary imaging, decrease costs, and prevent protracted throughput of patients at the ED in patients without an urgent condition. But it could also provide a timely and accurate diagnosis and management strategy in patients with an urgent condition. Such a test needs a high PPV and a low percentage of missed urgent cases besides specific features such as a wide availability, fast execution, and low costs.^21^ This study demonstrates that using CRP and WBC count as triage test alone would lead to an unacceptably high percentage of missed urgent cases and a substantial overshoot in use of diagnostic imaging because of the high percentage of false-positive cases.

Some studies have suggested that because of the properties of CRP, an acute-phase protein that can rise rapidly in case of an inflammation or infection, the duration of symptoms would be associated with the discriminatory capacity of CRP.^14^ Patients early in the onset of a disease could present with low values of inflammatory markers despite the underlying cause, whereas the chance of an urgent condition in patients with low values of inflammatory markers after a longer duration of symptoms would decrease. Nevertheless, our study demonstrates that the duration has only moderate influence on the accuracy of inflammatory markers. Even in patients with symptoms for >48 hours, CRP levels and WBC count have limited discriminative capacities.

An important limitation of our study is the fact that we only assessed CRP levels and WBC count as single predictors and not in combination with clinical parameters. In daily clinical practice, inflammatory markers are often combined in a diagnostic sequence. The probability of an urgent diagnosis is usually estimated combining the value of history and physical examination with inflammatory markers. Simply adding the diagnostic value of CRP and WBC count as assessed in this study on top of the diagnostic value of other tests such as history and physical examination would lead to an exaggeration of the diagnostic value of CRP levels and WBC count.^21^ Another limitation of our study was inherent to the designs of the studies used for this individual patient data meta-analysis. Preferably, diagnostic tests should be evaluated both in terms of patient outcome and diagnostic accuracy. In our study, however, insufficient data were available to analyze the effect on patient outcome. The classification of the final diagnosis into urgent and nonurgent conditions has a major influence on the diagnostic value of CRP and WBC. Using other classification systems, such as inflammatory versus noninflammatory diseases, might lead to a higher diagnostic accuracy of CRP levels and WBC count but is less clinically applicable. Not all inflammatory conditions need urgent treatment, whereas some noninflammatory conditions do need urgent treatment. We included all consecutive patients with acute abdominal pain presenting at the ED. Patients taking immunosuppressive drugs were not excluded, mimicking daily practice. This might have influenced the accuracy of CRP. An advantage of the present study design is the ability to analyze a large number of patients in combined study cohorts. This gives the study a greater power and makes it possible to draw more firm conclusions. Incorporating the complete range of values for CRP and WBC count in our analysis enabled us to analyze several clinically relevant cutoff values.

Future studies should aim at prospective assessment of the use of CRP levels and WBC count as a triage instrument for additional imaging, but evaluated in combination with all clinically relevant tests such as history and physical examination in a hierarchical manner closely mimicking daily practice. Studies have demonstrated that other biomarkers such as procalcitonin have a higher discriminatory value than CRP in diagnosing complicated acute appendicitis.^22^ These biomarkers could be assessed for their value as a triage instrument in patients with acute abdominal pain. An important factor that should be taken into account in these future studies is the time between onset of complaints and moment of determination of inflammatory parameters when assessing the diagnostic value.
